# Transcriptional Regulation and Function of Malic Enzyme 1 in Human Macrophage Activation

**DOI:** 10.3390/biomedicines12092089

**Published:** 2024-09-13

**Authors:** Anna Santarsiero, Simona Todisco, Paolo Convertini, Chiara De Leonibus, Vittoria Infantino

**Affiliations:** 1 Department of Science, University of Basilicata, 85100 Potenza, Italy; anna.santarsiero@unibas.it (A.S.); simona.todisco@unibas.it (S.T.); paolo.convertini@gmail.com (P.C.); 2 Department of Health Sciences, University of Basilicata, 85100 Potenza, Italy; chiara.deleonibus@unibas.it; 3 Telethon Institute of Genetics and Medicine (TIGEM), 80078 Pozzuoli, Italy

**Keywords:** malic enzyme 1, gene expression, NF-κB, macrophage activation, inflammation, immunometabolism, reactive oxygen species, nitric oxide, PGE2

## Abstract

Macrophages represent primary players of the innate immune system. Macrophage activation triggers several signaling pathways and is tightly associated with metabolic changes, which drive different immune subsets. Recent studies unveil the role of various metabolic enzymes in macrophage activation. Here, we show that malic enzyme 1 (ME1) is overexpressed in LPS-induced macrophages. Through chromatin immunoprecipitation, we demonstrate that ME1 transcriptional regulation is under control of NF-κB. Furthermore, ME1 activity is also increased in activated human PBMC-derived macrophages. Notably, ME1 gene silencing decreases nitric oxide as well as reactive oxygen species and prostaglandin E2 inflammatory mediators. Therefore, modulating ME1 provides a potential approach for immunometabolic regulation and in turn macrophage function.

## 1. Introduction

Malic enzyme (ME) catalyzes the reversible oxidative decarboxylation of L-malate into pyruvate and CO_2_ with the reduction of NAD (P)^+^ to NAD (P)H in the presence of a divalent metal ion, Mn^2+^ or Mg^2+^. This enzyme is widely distributed in all organisms, bacteria, plants, and animals. In humans, there are three isoforms of ME: cytosolic NADP^+^ ME (ME1), mitochondrial NAD (P)^+^ ME (ME2), and mitochondrial NADP^+^ ME (ME3). Structurally, MEs have the same homotetrameric quaternary structure, described as a double dimer with a separate active site in each monomer. ME2 isoform presents an allosteric site for the activator fumarate and binds to L-malate in a cooperative manner, whereas ME1 and ME3 are non-allosteric enzymes [1].

The function of ME1 in intermediary metabolism is well known, as it supplies cytosolic NADPH for lipogenesis and cholesterol biosynthesis and connects the glycolysis and tricarboxylic acid (TCA) cycle pathways through malate decarboxylation. As part of the citrate/pyruvate shuttle, ME1 plays a role in the homeostasis of reducing equivalents. The NADPH pool is mainly guaranteed in the cytoplasm by ME1, isocitrate dehydrogenase (IDH1), the one-carbon cycle, and the pentose phosphate pathway (PPP) [2], the regulation and crosstalk of which is still little known.

Different findings confirm a key function of ME1 in liver, adipose tissue, and in cancer cells. In this context, by balancing NADPH, it has been described as a crucial metabolic enzyme in regulating oxidative stress and biosynthesis of macromolecules [3]. Indeed, ME1 has been found increased in different kinds of cancers as well as IDH1 and glucose-6-phosphate-dehydrogenase (G6PD), likely preserving cancer cells from excessive reactive oxygen species (ROS) levels and in turn damage to molecules and apoptosis [4,5,6]. Furthermore, ME1 activates the PPP by interacting with 6-phosphogluconate dehydrogenase (6PGD), which is the second enzyme of this pathway, to provide NADPH [7]. Therefore, ME1 knockdown is linked to oxidative stress and NADPH decrease in cancer patients [6,8,9]. 

Macrophages play a major role in innate immunity by regulating inflammatory response and safeguarding tissue homeostasis. Heterogeneity and high plasticity are hallmarks of macrophages that show distinct phenotypes according to the microenvironmental signals. Toll-like receptor (TLR) ligands, including lipopolysaccharide (LPS), represent critical triggers during M1 proinflammatory macrophage polarization. Conversely, polarization into M2 macrophages—playing a central role in tissue remodeling and tumor promotion—is triggered by IL-4 and IL-13 [10]. M1 macrophages induce inflammatory responses and fight against infections through the production of inflammatory mediators as well as proinflammatory cytokines. However, if activated excessively and for a long time, proinflammatory macrophages may lead to tissue damage and sustained inflammation [11]. Moreover, M1 macrophages are involved in the development and progression of chronic inflammatory diseases [12], representing the dominant cellular population in the inflamed joint. Recently, it was demonstrated that M1 macrophages contribute to the initiation and progression of rheumatoid arthritis (RA) by inducing inflammation of synovial membrane and cartilage–pannus junction. Accordingly, M1 macrophages are the predominant immune cells found in RA synovium and a higher M1/M2 ratio is present in RA patients compared to healthy subjects [12].

More recently, several features of metabolic reprogramming have been emerging in immune cells. Macrophages regulate their activation, phenotype, and plasticity through various mechanisms, including the modulation of specific metabolic pathways [13,14]. Altered amino acid (including glutamine and arginine) and glycolytic and mitochondrial metabolism are hallmarks of the M1 macrophage subset as well as activated dendritic cells [15]. A growing number of studies highlight that metabolic rewiring controls inflammatory macrophages through regulating gene expression, levels of metabolites, and mediators of inflammation [16,17,18]. These findings have fostered investigations in immunometabolism. Among the immunometabolic pathways, the citrate metabolism has been recently evaluated. During classically macrophage activation with LPS or tumor necrosis factor alpha/interferon gamma (TNF-α/IFNγ), citrate is transported from the mitochondria to the cytosol via the mitochondrial citrate carrier (CIC). Once in the cytosol, citrate is cleaved by ATP citrate lyase (ACLY) into acetyl coenzyme A (acetyl-CoA) and oxaloacetate (OAA). Acetyl-CoA serves as a precursor for fatty acid or cholesterol biosynthesis, but it is also a substrate for histone and protein acetylation. OAA is reduced to malate by cytosolic malate dehydrogenase 1 (MDH1). Then, ME1 decarboxylates malate to pyruvate with production of NADPH. It is worth noting that NADPH is a substrate for both inducible nitric oxide synthase (iNOS) and NADPH oxidase (NOX), involved in the synthesis of nitric oxide (NO) and ROS inflammatory mediators, respectively. Furthermore, citrate-derived acetyl-CoA can be used for prostaglandin biosynthesis by supplying arachidonic acid for chain elongation of the essential fatty acid, linoleic acid.

We previously reported that both ACLY and SLC25A1—the gene coding CIC—are transcriptionally upregulated via NF-κB in LPS-activated macrophages. Interestingly, ACLY or CIC activity inhibition pulls down prostaglandin E2 (PGE2) secretion along with ROS and NO production in classically activated macrophages [16]. More recently, citrate-derived ACLY immunomodulatory activity has been linked to gene expression reprogramming through histone and NF-κB acetylation [19,20]. In vivo investigations indicate that ACLY knockdown in myeloid cell lineage improves atherosclerosis in mice [21]. Citrate pathway dysregulation has been found in Down syndrome, sepsis, and Behçet’s syndrome [19,22]. Furthermore, both ACLY and ME1 are found upregulated in PBMC-derived macrophages from metabolic dysfunction-associated steatohepatitis (MASH) patients [23]. An increase in malate levels has been observed upon 4 and 24 h LPS treatment of bone marrow-derived macrophages (BMDMs) [24]. These findings suggest a potential involvement of ME1 in macrophage activation. However, its role has been poorly investigated until now. Here, we demonstrate that *ME1* gene expression is upregulated in LPS-triggered macrophages. *ME1* gene promoter contains a phylogenetically conserved binding site for NF-κB that is active during macrophage activation. Accordingly, a significant increment in ME1 activity has been observed. Furthermore, inflammatory mediators such as TNF-α, NO, ROS, and PGE2 are decreased following *ME1* gene silencing in PBMC-derived macrophages triggered by LPS alone or LPS plus IFNγ.

In summary, our study provides new insights into the function of ME1 in macrophage regulation assessing its potential immunometabolic value and application in inflammatory diseases. 

## 2. Materials and Methods

### 2.1. Analysis of GEO Datasets for ME1 Expression

The Gene Expression Omnibus (GEO) (https://www.ncbi.nlm.nih.gov/geo/, accessed on 31 July 2024) was searched to understand whether *ME1* was differently expressed in patients suffering from immune-inflammatory disorders compared to healthy individuals. We analyzed GSE57383 dataset (gene expression of CD14^+^ cells from RA, PsA, and PsO patients with infliximab treatment) focusing on *ME1* gene expression from CD14^+^ primary cells from healthy controls and patients affected by RA at time point week 0, before starting an anti-TNF therapy. We further explored GEO dataset GSE82221 (GPL10558) by selecting normal controls and systemic lupus erythematosus (SLE) patients. The number of subjects and relative demographic features are listed in Table 1 (GSE57383) and Table 2 (GPL10558 series GSE82221).

GEO2R web tool (with R program operating back end) was used for comparisons between RA/SLE patients and healthy individuals in relative GEO series for differential *ME1* gene expression. Data are expressed as Log2 fold change between healthy individuals and RA/SLE patients.

### 2.2. Isolation of Human PBMCs from Whole-Blood and Differentiation of PBMC-Derived Macrophages

Peripheral blood mononuclear cells (PBMCs) were isolated from anonymized human whole-blood samples from healthy subjects. The informed consent was obtained from all participants. This study was carried out in agreement with the Declaration of Helsinki and the local Italian Committee on Human Research’s approved procedures (REF. TS/CEUR-CET/CEL n.20230044811—3 November 2023). Venous blood, collected into K2 EDTA-coated BD vacutainer tubes (Becton, Dickinson and Company, Franklin Lakes, NJ, USA), was mixed with Hanks’ Balanced Salt solution (HBSS, Sigma-Aldrich, St Louis, MO, USA) at a ratio of 1:2 (*v*/*v*). PBMCs were isolated by Histopaque-1077 (Sigma-Aldrich) density gradient centrifugation as previously described [25]. PBMCs were cultured for 3 days in Roswell Park Memorial Institute (RPMI) 1640 medium (Thermo Fisher Scientific, San Jose, CA, USA) supplemented with 100 ng/mL recombinant human M-CSF (Cell Guidance Systems, St. Louis, MO, USA), 10% fetal bovine serum, 2 mM L-glutamine, 100 U/mL penicillin, and 100 µg/mL streptomycin at 37 °C in a humidified chamber of 5% CO_2_. 

### 2.3. RNA Interference and Treatments

The *ME1* gene was transiently silenced via a specific small interfering RNA (siRNA) targeting human *ME1* (siME1, s8638, *Silencer^®^* Select, Thermo Fisher Scientific). PBMC-derived macrophages were transfected for two consecutive days with siME1 or Negative Control siRNA (4390843, Thermo Fisher Scientific) using Lipofectamine RNAiMax Reagent (13778-030, Thermo Fisher Scientific). Briefly, 2 µL of siRNA (25 μM) was mixed with 3 μL Lipofectamine^®^ RNAiMAX Reagent in 100 μL Opti-MEM Reduced Serum Medium (Thermo Fisher Scientific) and were incubated at room temperature for 15 min to form siRNA–Lipofectamine RNAiMAX complexes. The 100 μL transfection mixture was put into each well of a 12-well plate and 1 mL of cells (1 × 10^6^ cells) in complete growth medium was added. Twenty-four hours after the reverse transfection, the cells were transfected again. Cells were incubated at 37 °C and 5% CO_2_ for additional 24 h before LPS stimulation or other further treatments. 

The cells were incubated with 1 μg/mL LPS from *Escherichia coli* K12 (tlrl-peklps, Invivogen, CA, USA) alone or combined with 10 ng/mL IFNγ (ImmunoTools GmbH, Friesoythe, Germany) for up to 48 h. Where indicated, PBMC-derived macrophages were pretreated for 1 h with 20 μM IKK Inhibitor VII (IKK16, 401486, Sigma-Aldrich), 50 μM Apocynin (178385, Sigma-Aldrich), 20 μM Rotenone (R8875, Sigma-Aldrich), or 1 mM β-Nicotinamide adenine dinucleotide 2’-phosphate reduced tetrasodium salt hydrate (NADPH, N7505, Sigma-Aldrich) followed by stimulation with LPS.

### 2.4. Western Blot Analysis

The lysis of 2 × 10^6^ PBMC-derived macrophages was performed in 100 µL of an ice-cold 0.1% Nonidet P40/PBS solution carrying out three freeze–melt cycles (−80 °C for 8 min/40 °C for 4 min). After centrifugation at 8600× *g* at 4 °C for 5 min, the supernatant was collected. A Bradford assay (Pierce™ Coomassie (Bradford) Protein Assay Kit, 23200, Thermo Fisher Scientific) was used to determine protein concentration. A 4 × Laemmli Sample Buffer (1610747, Bio-Rad Laboratories, Hercules, CA, USA) was added to 30 µg of proteins, and the samples were boiled at 95° C for 5 min. The proteins were separated by 12% sodium dodecyl sulfate–polyacrylamide gel electrophoresis (SDS-PAGE) and transferred onto nitrocellulose membranes. The membranes were blocked for 1 h at room temperature in a tris-buffered saline solution containing 0.5% Tween (TBST) and 5% nonfat dry milk. Then, the membranes were immunostained overnight at 4 °C with anti-ME1 (ab97445, Abcam, Cambridge, MA, USA), anti-PPARγ (sc-7273, Santa Cruz Biotechnology, USA), anti-AMPKα1 (sc-398861, Santa Cruz Biotechnology), or anti-β–actin (ab8227, Abcam) primary antibodies. After three washes with TBST, the membranes were incubated for 1 h at room temperature with horseradish peroxidase (HRP) conjugated goat anti-rabbit secondary antibody (Santa Cruz Biotechnology, Santa Cruz, CA, USA). The immunoreactions were visualized at Chemidoc™ XRS detection system (Bio-Rad Laboratories) using WesternBright™ ECL (Advansta, Menlo Park, CA, USA). Image Lab software (Version 5.2.1) was used for image acquisition and densitometric analysis.

### 2.5. Quantitative Real-Time PCR (RT-qPCR)

Total RNA was extracted from 2 × 10^6^ cells using an RNeasy Plus Mini Kit (Qiagen, Hilden, Germany) as specified by the manufacturer. Complementary DNA (cDNA) was synthesized from 1 μg of total RNA by iScript™ cDNA Synthesis Kit (Bio-Rad Laboratories, Hercules, CA, USA) according to the following protocol: 5 min at 25 °C, 20 min at 46 °C, and 1 min at 95 °C. RT-qPCR experiments were run in triplicate with human ME1 (Hs00159110, RefSeq NM_002395.5) and β-actin (Hs01060665, RefSeq NM_001101.3) TaqMan Gene Expression Assays (Thermo Fisher Scientific) on the 7500 Fast Real-Time PCR System (Thermo Fisher Scientific). Data were analyzed according to the ΔΔCt method, as previously reported [26]. The fold changes in ME1 expression were determined as 2^−ΔΔCt^.

### 2.6. ME1 Activity

ME1 activity was assayed in cell extracts following the oxidative decarboxylation of L-malate by the spectrophotometric method based on the increase in absorbance of NADPH at 340 nm. PBMC-derived macrophages (1 × 10^7^), treated for 0–1–2–4–8 h with LPS, were collected and washed twice in ice-cold PBS by centrifugation at 8600× *g* at 4 °C for 5 min. The cell pellet was resuspended in an ice-cold 0.1% Nonidet P40/PBS solution, and three freeze–melt cycles (−80 °C for 8 min/40 °C for 4 min) were carried out. The supernatant was recovered after centrifugation at 8600× *g* at 4 °C for 5 min, and the protein concentration was measured by a Bradford assay. To determine ME1 activity, 150 µg of proteins were added to the standard assay mixture made of 50 mM Tris/HCl (pH 7.4), 0.5 mM NADP^+^ (Sigma-Aldrich), and 1 mM MnCI_2_. The reaction was started by adding 10 mM L-malate (Sigma-Aldrich) and followed by tracking the increase in NADPH absorbance at 340 nm at 25 °C for 20 min with a spectrophotometer (Multiskan Sky, Thermo Fisher Scientific). The specific ME1 activity was normalized to the protein concentration and expressed as a percentage of the control (set at 100%).

### 2.7. In Silico Analysis of ME1 Promoters

Gene sequences of *ME1* genes from *Homo sapiens*, *Mus musculus*, *Rattus norvegicus*, and *Sus scrofa* were retrieved as FASTA file from National Center for Biotechnology Information (NCBI) Genome Browser (https://www.ncbi.nlm.nih.gov/gene, accessed on 31 July 2024) and used for in silico analysis. Promoter regions were defined as the 2 kb upstream sequence from the start codon of each gene. AliBaba 2.1 software (http://gene-regulation.com/pub/programs/alibaba2, accessed on 31 July 2024) was employed to predict the potential binding sites of transcription factors within the *ME1* promoter region.

### 2.8. ChIP-qPCR 

Human PBMC-derived macrophages (5 × 10^6^), at the end of treatment with LPS for 3 h, were fixed by 1% formaldehyde at 37 °C for 10 min. Subsequently, the cells were collected by centrifugation and the cell pellet was resuspended in a lysis buffer (5 mM Pipes pH 8.0, 85 mM KCl, 0.5% NP40, 1 × PIC). The cells were lysed by 10 strokes of a chilled Potter-Elvehjem homogenizer and sheared by sonication at 70% power, cycle 9, for 10 min in a 0.1% SDS lysis buffer to generate chromatin fragments of 300–400 bp. The chromatin was immunoprecipitated overnight at 4 °C on a rocking platform using 5 µg of anti-NF-κB/p65 specific antibody (ab16502, Abcam) and 40 µL A/G PLUS agarose beads (SC-2003, Santa Cruz). After reverse cross-linking with RNase and Protease K, the immunoprecipitates were purified using a PureLink™ PCR Purification Kit (Thermo Fisher Scientific) and analyzed by qPCR using SYBR™ Green PCR Master Mix (Thermo Fisher Scientific) and 20 pmol forward (5′-CACCCTAACACCTTGACTCTTATC-3′) and reverse (5′-CCCTCTTTCTACAGATCCACAAC-3′) primers suitable to amplify the 227 bp region of the human *ME1* promoter containing the NF-κB binding site. The reactions were run on a 7500 Fast Real-Time PCR System (Thermo Fisher Scientific). 

### 2.9. ROS and NO Detection 

The quantification of reactive oxygen species (ROS) and nitric oxide (NO) levels was performed on PBMC-derived macrophages (1 × 10^6^) after 24 h stimulation with LPS or LPS + IFN γ. Where indicated, apocynin, rotenone, and NADPH were added 1 h before LPS. ROS and NO concentrations were determined by using fluorescent probes 6-carboxy-2′, 7′-dichlorodihydrofluorescein diacetate (DCF-DA, Thermo Fisher Scientific, San Jose, CA, USA) and 4-amino-5-methylamino-2′, 7′-difluorofluorescein diacetate (DAF-FM diacetate, Thermo Fisher Scientific), respectively. In detail, cells were collected, counted, and centrifuged at 6500 × *g* for 5 min. The cell pellet was resuspended in PBS to have 10^5^ cells/100 µL. In cell suspension, 10 µM DCF-DA or 2.24 µM DAF-FM diacetate were added. After incubation in the dark at 37 °C for 30 min, 100 µL of the sample was transferred to triplicate wells on a black microtiter plate. Fluorescence was measured using a GloMax^®^ Discover Microplate Reader (Promega, Madison, WI, USA). 

### 2.10. PGE2 and TNF-α Detection 

PBMC-derived macrophages (5 × 10^5^) were treated with LPS or LPS + IFN γ following ME1 gene silencing. PGE2 levels were measured after 48 h of LPS or LPS + IFN-γ treatment using a DetectX^®^ Prostaglandin E2 High-Sensitivity Immunoassay Kit (Arbor Assays, Ann Arbor, MI, USA) as previously reported [25]. 

TNF-α levels were quantified after 24 h of LPS treatment using a commercially available ELISA kit (Immunotools, Friesoythe, Germany) following the manufacturer’s instructions.

### 2.11. Statistical Analysis

Statistical analysis was performed using GraphPad Prism software version number 8.0.2 (La Jolla, CA, USA). Results are presented as mean ± standard deviation (SD) from three independent experiments, each run at least in triplicate. For pairwise comparisons, we used Student’s *t*-test when the data were normally distributed. If the data did not follow a normal distribution, we employed the non-parametric Mann–Whitney U test. One-way ANOVA was utilized for comparisons involving more than two groups. Tukey’s test was used for post hoc analysis to compare every mean with every other mean. When Tukey’s post-hoc test was performed, we used different letters in the figures to indicate significant differences between treatments at *p* < 0.05. Dunnett’s test was chosen as an alternative when we tested for differences only in comparison to unstimulated cells. The statistical method employed for each experiment is detailed in the figure legends. The asterisks in the figures indicate statistical significance (* *p* < 0.05; ** *p* < 0.01; and *** *p* < 0.001). 

## 3. Results

### 3.1. ME1 Expression in Rheumatoid Arthritis and Systemic Lupus Erythematosus

**Figure 1:**
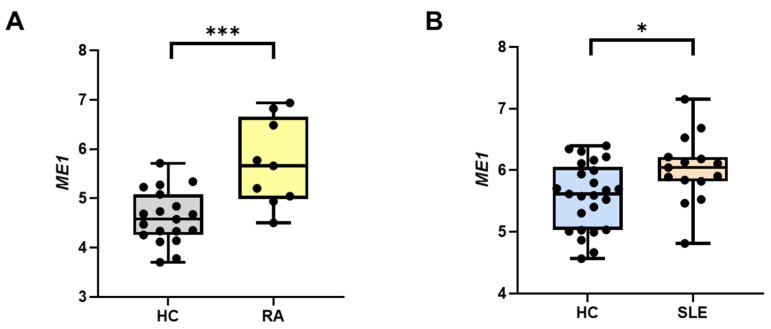
ME1 expression levels in rheumatoid arthritis and systemic lupus erythematosus: (**A**) Box plot with individual data points for ME1 gene expression in peripheral blood CD14^+^ monocytes from healthy controls (HC) and rheumatoid arthritis (RA) patients from published array GSE57383. (**B**) Box plot with individual data points for ME1 gene expression in peripheral blood mononuclear cells from healthy subjects (HC) and systemic lupus erythematosus (SLE) patients from GEO dataset GSE82221 (GPL10558). Statistical analysis was performed by using a two-tailed unpaired Mann–Whitney U test (* *p* < 0.05, *** *p* < 0.001).

Considering the recent findings highlighting the role of the citrate export pathway in macrophage activation and inflammation, we wondered if ME1—a citrate–pyruvate shuttle enzyme—could also be involved in innate immune regulation. To this end, we performed a preliminary analysis of disease gene expression databases to establish whether there were differences in ME1 expression in patients affected by chronic inflammatory diseases. Analysis of GEO dataset GSE57383 revealed a significant upregulation of ME1 in CD14^+^ monocytes from patients affected by RA (mean ± SD = 5.71 ± 0.87) in comparison to healthy individuals (mean ± SD = 4.61 ± 0.54) (Figure 1A). We also observed a slight but significant increase in ME1 gene expression in PBMCs derived from patients with SLE (mean ± SD = 6.02 ± 0.55) versus healthy controls (mean ± SD = 5.58 ± 0.54) (Figure 1B) when we examined the GSE82221 (GPL10558) dataset. These data suggest a potential involvement of ME1 in inflammatory diseases.

### 3.2. Early Activation of ME1 in LPS-Treated Human Macrophages

**Figure 2:**
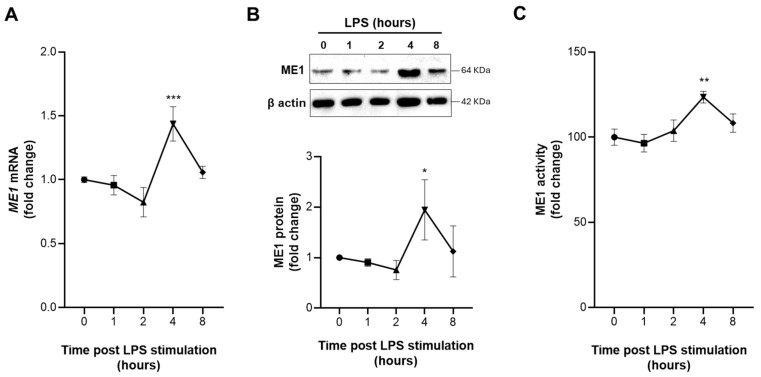
Induction of ME1 expression and activity in LPS-stimulated human macrophages. Human PBMC-derived macrophages were triggered by LPS, and ME1 mRNA (**A**), protein levels (**B**), and activity (**C**) were evaluated over time. Panel (**B**) shows representative Western blotting from 3 independent experiments, with protein levels quantified relative to β-actin and normalized to untreated cells (0). The bottom panel in (**B**) displays ME1 protein quantitation across the 3 independent Western blots. In (**C**), the ME1 activity is expressed as a percentage compared to the control (0). In (**A**,**C**) data are presented as means ± SD (error bars) from 3 independent experiments. Significant differences, where indicated in (**A**–**C**), were determined using one-way ANOVA followed by Dunnett’s multiple comparison test (* *p* < 0.05; ** *p* < 0.001, *** *p* < 0.001).

In order to investigate the role of ME1 in the metabolic reprogramming of innate immune cells, we performed a time course of both mRNA and protein levels in human PBMC-derived macrophages triggered by LPS. Real-time PCR experiments showed a significant increase in *ME1* mRNA levels after 4 h of LPS treatment. Accordingly, through Western blotting analysis, we observed a significant increase in the ME1 protein amount at the same time (Figure 2A,B). Remarkably, the ME1 enzymatic activity time course showed a fast and strong peak of activation following 4 h activation with LPS (Figure 2C). To confirm the specificity for the M1 program, protein levels of PPARγ and AMPKα1, two well-known markers of M2 macrophages [27,28], were evaluated. Supplementary Figure S1 shows a marked decrease in PPARγ and a slight reduction in AMPKα1 in LPS-activated macrophages. All together, these results suggest an early involvement of ME1 in inflammatory signaling of classically activated human macrophages. 

### 3.3. Identification and Functionality of the NF-κB-Responsive Element in ME1 Gene Promoter 

**Figure 3:**
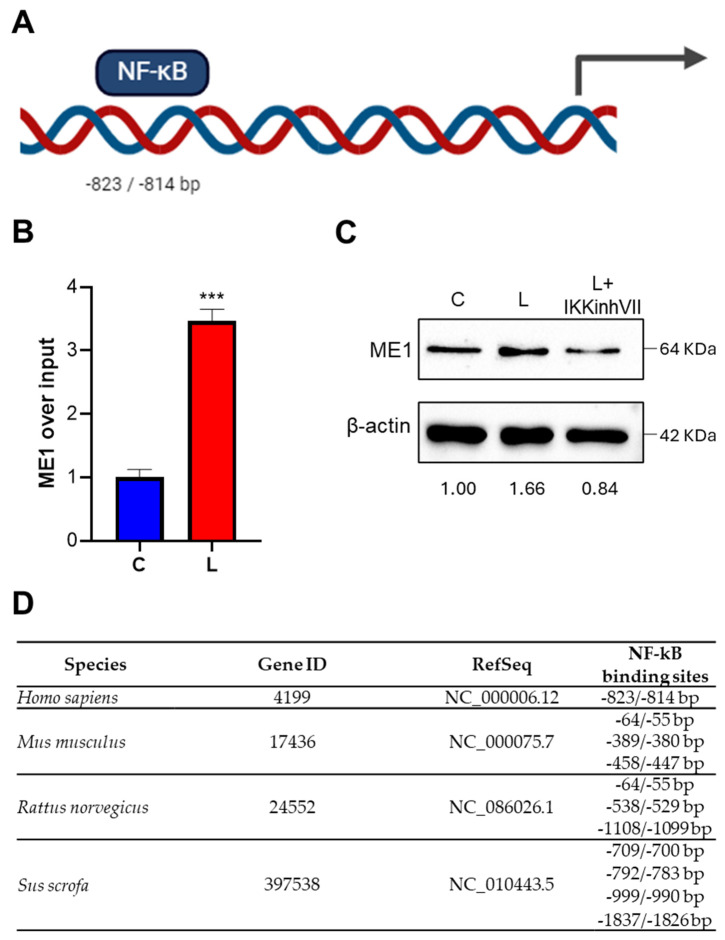
Effect of NF-κB on ME1 gene expression. (**A**) Schematic representation showing NF-κB-responsive element location in the human ME1 gene promoter. (**B**) Human PBMC-derived macrophages untreated (**C**) or triggered by LPS (L) were used to perform chromatin immunoprecipitation (ChIP) analysis with an antibody against p65 (subunit of NF-κB). After immunoprecipitation, ME1 gene promoter was analyzed in real-time PCR experiments by specific primers. Differences were significant according to Student’s *t*-test (*** *p* < 0.001). (**C**) The same cells, indicated as C and L, in the presence or absence of IKK inhibitor VII (IKKinhVII) were analyzed by Western blotting with a specific anti-ME1 antibody. Protein levels are quantified against β-actin. Western blots presented are representative of 3 experiments. Means of protein values were normalized versus the mean of protein in control macrophages (**C**). (**D**) Panel displaying the location of NF-κB-responsive elements in promoter of mammals of the following species: *Homo sapiens*, *Mus musculus*, *Rattus norvegicus*, and *Sus scrofa*. Gene ID and RefSeq of each sequence are also reported.

Since we found high levels of ME1 gene expression and activity in classically activated macrophages, we wondered what molecular mechanisms were underlying this effect. To this end, we performed an in silico analysis of the human ME1 gene promoter. This analysis revealed the presence of an NF-κB-responsive element located at −823/−814 bp upstream of the translational start site (Figure 3A). In order to check if the NF-κB binding site was active in our experimental conditions, ChIP experiments were carried out by using a p65 specific antibody, because it is well known that p50/p65 heterodimer represents the main form of NF-κB in M1 proinflammatory macrophages [29]. Figure 3B shows that LPS stimulation of PBMC-derived macrophages induced a strong binding of NF-κB to the human ME1 gene promoter. Interestingly, the inhibition of NF-κB signaling through IKK inhibitor VII markedly reduced ME1 protein content (Figure 3C). Furthermore, the presence of NF-κB-responsive elements in other mammals (Figure 3D) demonstrates how important this transcriptional factor is in regulating ME1 gene expression. Our data so far indicate that the upregulation of the human ME1 gene is under control of NF-κB in classically activated macrophages.

### 3.4. Effect of ME1 Gene Silencing on NO and PGE2 Secretion

**Figure 4:**
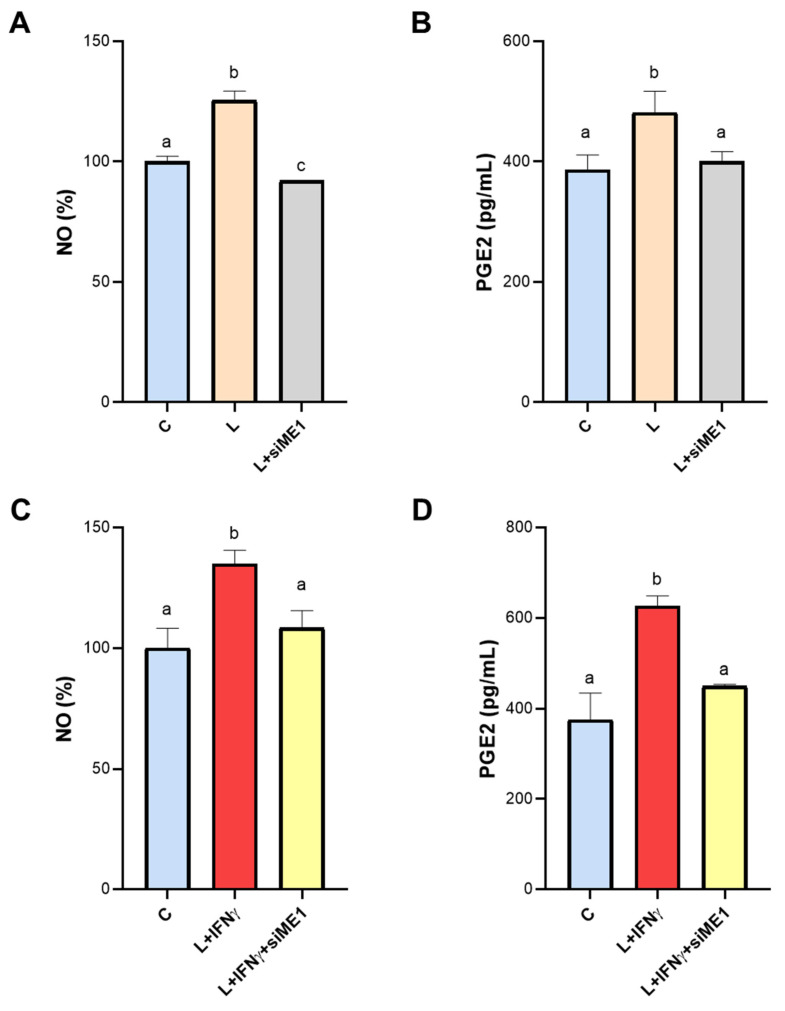
NO and PGE2 production in *ME1*-silenced macrophages. Human PBMC-derived macrophages were transfected for two consecutive days with a specific siRNA targeting human ME1 (siME1) or negative control siRNA (C). At twenty-four hours from the last gene silencing, cells were triggered by LPS (L) (**A**,**B**) or LPS + IFNγ (L+ IFNγ, C and D). NO (**A**–**C**) and PGE2 (**B**–**D**) levels were determined following 24 and 48 h of LPS ± IFNγ stimulation, respectively. Data are presented as means ± SD (error bars) of 3 independent experiments, each with at least three replicates. Letters above the bars (a, b, and c) refer to statistical analysis performed by one-way ANOVA followed by Tukey’s post-hoc test: different letters indicate significant differences between treatments at *p* < 0.05.

Inducible nitric oxide synthase becomes active when macrophages are exposed to inflammatory stimuli, such as LPS, and is responsible for huge NO generation. It is worth noting that NADPH is essential for iNOS activity. Indeed, the reaction of conversion of L-arginine to NO and L-citrulline, catalyzed by iNOS, requires NADPH as cofactor. In human LPS-triggered macrophages, *ME1* gene silencing caused a drastic reduction in NO levels, bringing levels back to baseline (Figure 4A). A similar trend was observed when we quantified PGE2: in the presence of the specific siRNA against the human ME1 gene, PGE2 content was similar to those of unstimulated cells (C) (Figure 4B). 

Synergistic induction by LPS and IFN-γ is known to strengthen the activation of the proinflammatory macrophage phenotype via canonical, inflammatory NF-κB target genes [30]. Therefore, we performed the same experiments in the presence of both LPS and IFN-γ. Indeed, we observed a more marked secretion of the proinflammatory mediators in macrophages induced by LPS + IFNγ: a 35% increase in NO and almost 70% increase in PGE2 compared to the control (Figure 4C,D). Even under these conditions, *ME1* gene silencing lowered NO and PGE2 levels. In the case of NO, we observed a significant reduction of 20% compared to cells treated with LPS + IFN-γ (Figure 4C). For PGE2, the shift was like that observed in cells activated by LPS alone, with levels being restored to control values (Figure 4D). The comparison between NO and PGE2 levels in untreated macrophages (Unt MP) and macrophages transfected with negative control siRNA (C) rule out any off-target effect (Supplementary Figure S2A,B).

Altogether, our findings suggest that NADPH produced by ME1 is crucial for NO and PGE2 secretion during M1-like state macrophage activation since human *ME1* gene silencing abrogates the increase of both these inflammatory mediators upon LPS addition. 

### 3.5. ROS Generation Mediated by ME1 in M1-like State Macrophages

**Figure 5:**
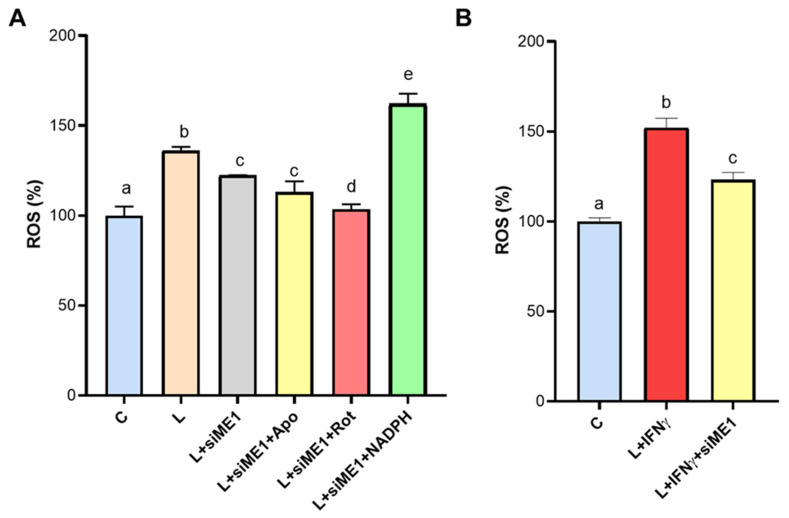
ROS generation is mediated by ME1 activity. Human PBMC-derived macrophages were transfected for two consecutive days with a specific siRNA targeting human ME1 (siME1) or negative control siRNA (C). At twenty-four hours from the last gene silencing, cells were triggered by LPS (L, **A**) or LPS + IFNγ (L+ IFNγ, **B**). Where indicated, cells were pretreated for 1 h with 50 μM apocynin (L+siME1+Apo), 20 μM rotenone (L + siME1 + Rot), or 1 mM NADPH (L + siME1 + NADPH). After 24 h treatment, ROS were measured. Data are expressed as the mean ± SD (error bars) of three independent experiments. Letters above the bars (a, b, c, d, and e) refer to results from one-way ANOVA followed by Tukey’s post-hoc test: different letters indicate significant differences between treatments at *p* < 0.05.

To ascertain the role of ME1 in the production of ROS in proinflammatory macrophages, we measured ROS levels in *ME1*-silenced PBMC-derived macrophages triggered by LPS or LPS + IFNγ. The combination of LPS and IFNγ led to greater ROS secretion (152.1 ± 5.2 % of C, Figure 5B) compared to cells activated with LPS alone (136.1 ± 2.1 % of C, Figure 5A). *ME1* gene silencing reduced ROS levels in both cases (Figure 5A,B). Any off-target effects can be ruled out, as ROS levels in macrophages treated with negative control siRNA were comparable to those in untreated cells (Supplementary Figure S2C). Given that NOX and the mitochondrial respiratory chain may be the primary sources of ROS under these conditions, we measured ROS levels in the presence or absence of apocynin (the NOX inhibitor) and rotenone (inhibitor of the mitochondrial respiratory chain Complex I). Figure 5A illustrates how ME1-silenced PBMC-derived macrophages displayed reduced ROS levels that were unaffected by apocynin compared to LPS treatment. Conversely, ROS levels in the presence of rotenone were the same as those of control (Figure 5A) suggesting that mitochondrial ROS production could be ME1-independent in macrophages. Remarkably, high levels of ROS were observed when NADPH was added, by highlighting the involvement of ME1-derived NADPH in ROS generation in classically activated human macrophages. 

### 3.6. Effect of ME1 Gene Silencing on TNF-α Secretion 

It is well-established that TNF-α, a key inflammatory mediator involved in the pathogenesis of numerous diseases, including RA and SLE, is regulated by NF-κB [31]. In RA, TNF-α is the major inflammatory cytokine driving disease progression and joint inflammation [32]. Likewise, increased serum levels of TNF-α in SLE patients correlate with disease activity and are linked to systemic manifestations, including cardiovascular disease and lupus nephritis [33]. Furthermore, the secretion of TNF-α by human macrophages is notably induced by LPS stimulation [31].

**Figure 6:**
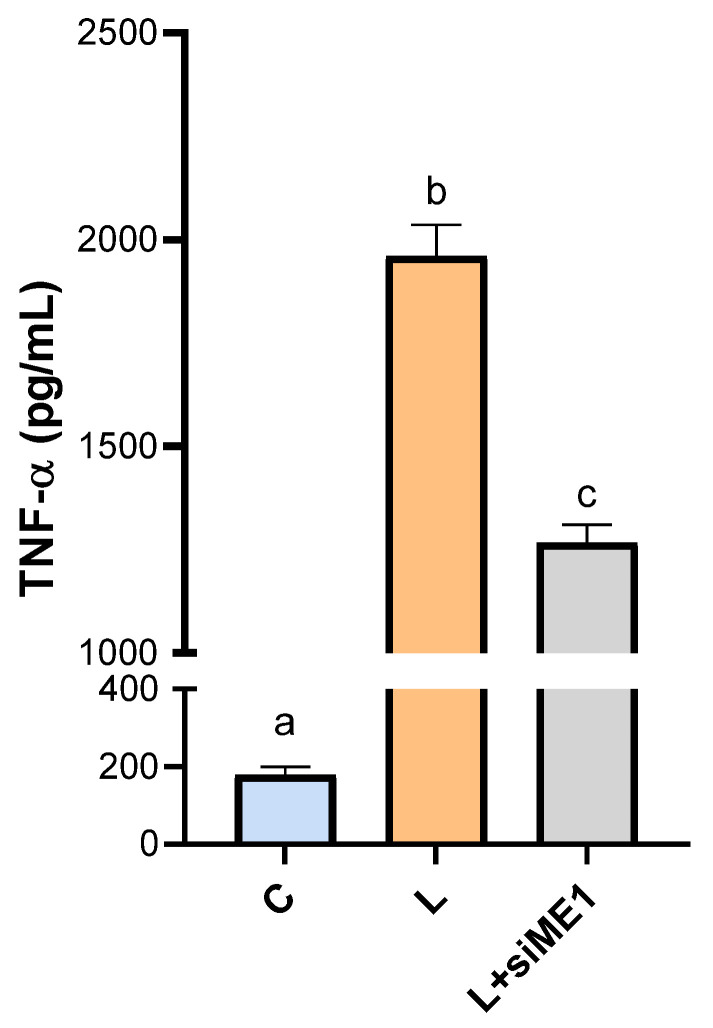
TNF-α production in *ME1*-silenced macrophages. Human PBMC-derived macrophages were transfected for two consecutive days with a specific siRNA targeting human ME1 (siME1) or negative control siRNA (C). At twenty-four hours from the last gene silencing, cells were triggered by LPS (L). TNF-α levels were determined following 24 h of LPS stimulation. Data are presented as means ± SD (error bars) of 3 independent experiments with at least three replicates in each. Letters above the bars (a, b, and c) refer to statistical analysis performed by one-way ANOVA followed by Tukey’s post-hoc test: different letters indicate significant differences between treatments at *p* < 0.05.

Considering that (i) ME1 is overexpressed in RA and SLE patients, (ii) ME1 is upregulated in macrophages activated by LPS, and (iii) ME1 is under the transcriptional control of NF-kB, we measured the levels of TNF-α following *ME1* gene silencing. Interestingly, we observed a significant decrease in TNF-α levels in macrophages where ME1 was silenced, compared to those treated with LPS alone (Figure 6). These results suggest a crucial interaction between NF-κB, TNF-α, and ME1, highlighting the potential regulatory role of ME1 in the inflammatory response mediated by NF-κB and TNF-α. Our findings open avenues for further investigation into the molecular mechanisms governing inflammatory responses and the potential therapeutic targeting of ME1 in inflammatory diseases.

## 4. Discussion

Main functions of the macrophages are played in development, homeostasis, innate immunity, and inflammation. As a result, macrophage dysfunctions are involved in a large number of diseases, such as chronic obstructive pulmonary disease (COPD), atherosclerosis, autoimmune diseases, asthma, and coronavirus pneumonia COVID-19 [14,34,35].

A hallmark of macrophages is the plasticity referring to their ability to change function, features, and phenotype through polarization in response to external or internal triggers. A complex network of signaling cascades links stimulation to metabolic and gene expression reprogramming resulting in different subsets. 

Among transduction mechanisms, the canonical NF-κB activation pathway fosters the transcription of numberless proinflammatory genes in classically activated macrophages. In the last few years, it has been reported that metabolic signals including AMP-activated protein kinase (AMPK), ACLY, succinate, and peroxisome receptors PPARα and PPARγ control NF-κB activity in innate immune cells [36,37]. Therefore, immunometabolic crosstalk between NF-κB signaling and metabolism emerges in macrophage activation. Here, we demonstrate that *ME1* gene expression is upregulated in classically activated macrophages through NF-κB, whose signaling inhibition abolishes the effect. Furthermore, ChIP experiments reveal an increased binding of NF-κB to the human *ME1* gene promoter following LPS stimulation. Interestingly, a responsive element for NF-κB is also present in *ME1* gene promoter of other mammals such as *Mus musculus*, *Sus scrofa*, and *Rattus norvegicus*. 

Our in silico analysis unveils a significant upregulation of *ME1* gene expression in rheumatoid arthritis and lupus erythematosus highlighting a potential role of ME1 in inflammatory diseases. Proinflammatory signaling activates NOX, which generates superoxide anion (O_2_^•−^) in an NADPH-dependent manner. Accordingly, NOX activity is dysregulated in autoimmune diseases and injury caused by infections [38]. Mechanistically, we found a relationship between ME1-derived NADPH and NOX activity. Indeed, *ME1* gene silencing reduces ROS, and the presence of NADPH nullifies the decrement, while apocynin (NOX inhibitor) addition does not affect the trend in human-activated macrophages. 

Mitochondria represent another central source of ROS as metabolic byproducts during oxidative phosphorylation [39]. It has been another central source observed that mitochondrial ROS foster pyrin domain containing 3 (NLRP3) inflammasome activation through different molecular mechanisms including the cytosolic release of mitochondrial DNA (mtDNA) which interacts directly with NLRP3 [40]. Notably, inhibition of the mitochondrial complex I of the electron transport chain, a major source of mitochondrial ROS, significantly reduces ROS levels in *ME1*-silenced human macrophages. Therefore, mitochondrial ROS production seems ME1-independent in macrophages. Conceptually, these findings demonstrate a link between ME1 and NOX activity to drive the production of O2^•−^, a key inflammatory mediator. 

NADPH is also required as cofactor for inducible nitric oxide synthase (iNOS or NOS2) and in turn for NO biosynthesis in LPS- or cytokine-triggered macrophages. Nuclear factor 1 transducer and activator of transcription 1a (STAT-1a) and NF-κB signaling activation mediates transcriptional upregulation of iNOS. As critical inflammatory mediator, increased levels of exhaled NO are present in asthmatics, while their lowering follows treatment with corticosteroids [41]. Upregulation of iNOS has been found in allergic asthma and exhaled NO measurements have currently become clinical routine in disease management [42]. Moreover, measuring mucosal release of NO is a method to detect inflammatory processes in inflammatory bowel disease, vaginitis, and cystitis [43]. Our observations prove that iNOS activity is also dependent on ME1 activity since ME1 gene silencing reduces NO levels. 

However, the role of ME1 seems much wider as *ME1* gene silencing significantly affects PGE2 secretion, likely through supplying cytosolic NADPH for arachidonic acid biosynthesis [44]. It is worth noting that the significant decrease in ROS, NO, and PGE2 in LPS + IFNγ-activated and *ME1*-silenced macrophages strengthens the role of ME1 in proinflammatory M1 macrophages. Therefore, our findings bring to light ME1 as a new immunometabolic enzyme target of NF-κB signaling in innate immune cells. We can speculate that overexpression and activation of ME1 in LPS-activated macrophages contributes to the “immuno-NADPH” pool, i.e., the NADPH pool is necessary to activate the inflammatory response through NOX, iNOS, and prostaglandin biosynthesis. 

Future investigations to delve deeper into molecular mechanisms underlying ME1 in inflammatory diseases as well as further studies about specific ME1 inhibitors will be important in understanding ME1 immunometabolic role in immune cells. 

## Figures and Tables

**Table 1:** Demographic features of subjects analyzed from GSE57383 dataset.

	HC (n = 19)	RA (n = 9)
Age, mean ± SD (years)	42.0 ± 13.3	50.8 ± 11.1
Male sex, num (%)	6 (32)	1 (11)
Female sex, num (%)	13 (68)	8 (89)

Abbreviations: HC = healthy control; RA = rheumatoid arthritis.

**Table 2:** Demographic features of subjects analyzed from GSE82221 (GPL10558) dataset.

	HC (n = 25)	SLE (n = 15)
Age, mean ± SD (years)	32.2 ± 9.0	28.0 ± 8.7
Male sex, num (%)	5 (20)	2 (13)
Female sex, num (%)	20 (80)	13 (87)

Abbreviations: HC = healthy control; SLE = systemic lupus erythematosus.

## Data Availability

The data used to support the findings of this study are available from the corresponding authors upon request.

## Supplementary Materials

The following are available online at https://www.mdpi.com/article/10.3390/biomedicines12092089/s1, Figure S1: Specificity for the M1 program, Figure S2: NO⋅, PGE2, and ROS levels in untreated macrophages and macrophages transfected with negative control siRNA.
