# Supplementary material for: Transcriptional Regulation and Function of Malic Enzyme 1 in Human Macrophage Activation
Source: Biomedicines. 2024 Sep 13;12(9):2089. doi: 10.3390/biomedicines12092089 (PMC11428690; doi:10.3390/biomedicines12092089)
Supplement: Supplementary file 1 [file biomedicines-12-02089-s001.zip › biomedicines-3162898-supplementary.pdf]

Supplementary Materials

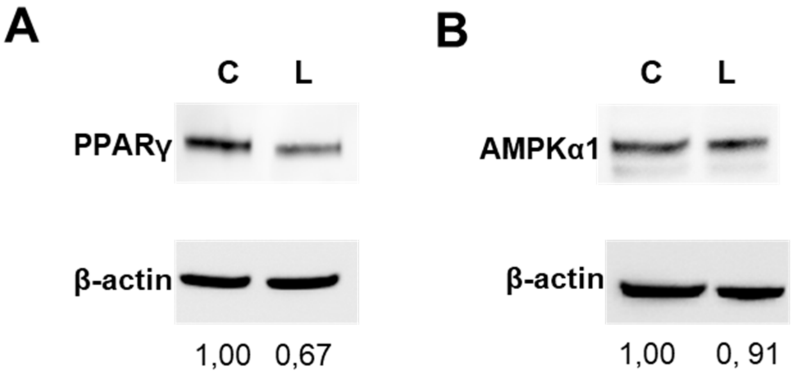

Figure S1. Specificity for the M1 program. Human PBMC-derived macrophages were triggered by LPS (L), and protein levels of two gene signatures of M2 macrophages, PPARγ (**A**) and AMPKα1 (**B**), were quantified. Protein levels were quantified against β-actin and normalized versus untreated cells (C).

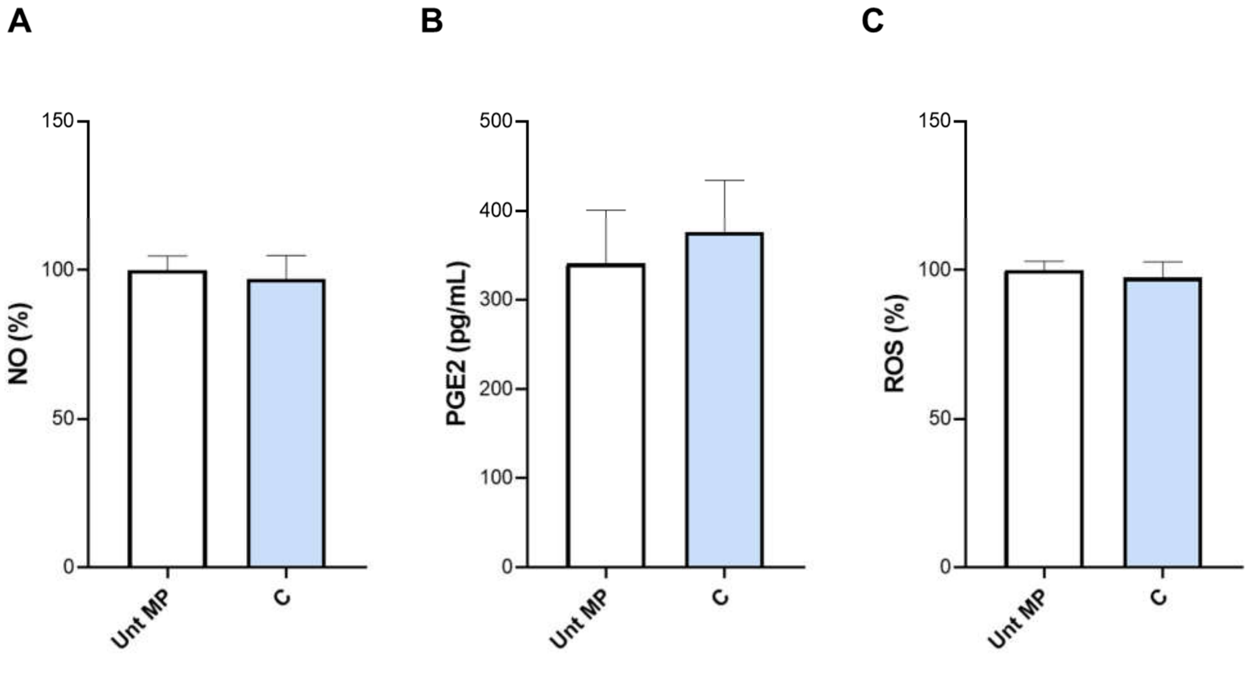

Figure S2. NO·, PGE2, and ROS levels in untreated macrophages (Unt MP) and macrophages transfected with negative control siRNA (C). Human PBMC-derived macrophages were transfected for two consecutive days with negative control siRNA (C). Untreated cells (Unt MP) were used as controls. NO· (**A**), PGE2 (**B**), and ROS (**C**) levels were determined following 48 hours of transfection. Data are presented as means ± SD (error bars) of 3 independent experiments with at least three replicates in each. Differences were not statistically significant according to Student's t-test.
